# Clinical effectiveness and cost-effectiveness of the Rehabilitation Enablement in Chronic Heart Failure (REACH-HF) facilitated self-care rehabilitation intervention in heart failure patients and caregivers: rationale and protocol for a multicentre randomised controlled trial

**DOI:** 10.1136/bmjopen-2015-009994

**Published:** 2015-12-23

**Authors:** R S Taylor, C Hayward, V Eyre, J Austin, R Davies, P Doherty, K Jolly, J Wingham, R Van Lingen, C Abraham, C Green, FC Warren, N Britten, C J Greaves, S Singh, S Buckingham, K Paul, H Dalal

**Affiliations:** 1 Institute of Health Research, University of Exeter Medical School, Exeter, UK; 2 Peninsula Clinical Trials Unit, Plymouth University, Plymouth, Devon, UK; 3 Heart Failure Services and Cardiac Rehabilitation, Aneurin Bevan University Health Board, Nevill Hall Hospital, Abergavenny, UK; 4 Cardiology Department, Sandwell & West Birmingham Hospitals NHS Trust, Birmingham, UK; 5 Department of Health Sciences, University of York, York, UK; 6 Institute for Applied Health Research, University of Birmingham, Birmingham, UK; 7 Department of Research, Development & Innovation, Royal Cornwall Hospitals NHS Trust, Truro, UK; 8 Cardiology Department, Royal Cornwall Hospitals NHS Trust, Truro, UK; 9 Psychology Applied to Health Group, University of Exeter Medical School, Exeter, UK; 10 Centre for Exercise and Rehabilitation Science, University Hospitals of Leicester NHS Trust, Glenfield Hospital, Leicester, UK; 11 REACH-HF Patient and Public Involvement Group, c/o Research, Development & Innovation, Royal Cornwall Hospitals NHS Trust, Truro, UK

**Keywords:** REHABILITATION MEDICINE

## Abstract

**Introduction:**

The Rehabilitation EnAblement in CHronic Heart Failure (REACH-HF) trial is part of a research programme designed to develop and evaluate a health professional facilitated, home-based, self-help rehabilitation intervention to improve self-care and health-related quality of life in people with heart failure and their caregivers. The trial will assess the clinical effectiveness and cost-effectiveness of the REACH-HF intervention in patients with systolic heart failure and impact on the outcomes of their caregivers.

**Methods and analysis:**

A parallel two group randomised controlled trial with 1:1 individual allocation to the REACH-HF intervention plus usual care (intervention group) or usual care alone (control group) in 216 patients with systolic heart failure (ejection fraction <45%) and their caregivers. The intervention comprises a self-help manual delivered by specially trained facilitators over a 12-week period. The primary outcome measure is patients’ disease-specific health-related quality of life measured using the Minnesota Living with Heart Failure questionnaire at 12 months’ follow-up. Secondary outcomes include survival and heart failure related hospitalisation, blood biomarkers, psychological well-being, exercise capacity, physical activity, other measures of quality of life, patient safety and the quality of life, psychological well-being and perceived burden of caregivers at 4, 6 and 12 months’ follow-up. A process evaluation will assess fidelity of intervention delivery and explore potential mediators and moderators of changes in health-related quality of life in intervention and control group patients. Qualitative studies will describe patient and caregiver experiences of the intervention. An economic evaluation will estimate the cost-effectiveness of the REACH-HF intervention plus usual care versus usual care alone in patients with systolic heart failure.

**Ethics and dissemination:**

The study is approved by the North West—Lancaster Research Ethics Committee (ref 14/NW/1351). Findings will be disseminated via journals and presentations to publicise the research to clinicians, commissioners and service users.

**Trial registration number:**

ISRCTN86234930; Pre-results.

## Introduction

Heart failure (HF) is a generally progressive condition that is estimated to affect 900 000 people in UK^1^ and is associated with significant health expenditure, amounting to around 1.0–3.2% of the total healthcare expenditure in Western Europe, North America and Latin America.^2^

People with HF experience a range of symptoms including shortness of breath at rest or on exertion, fatigue, fluid retention, impaired cognitive function and appetite disturbance.^3^ ^4^ HF is categorised as either HF with reduced ejection fraction (also known as systolic HF or left ventricular systolic dysfunction), or HF with preserved ejection fraction (also known as diastolic HF). Systolic HF is due to impaired left ventricular contraction, which results in a reduced ejection fraction (usually <45%) and diastolic HF is due to stiffness of the ventricle wall delaying filling of the heart chamber.^5^

Advances in pharmacological therapies and devices (implantable cardioverter defibrillators and biventricular pacing) have been shown to improve physiological parameters and quality of life, reduce symptoms and decrease mortality and readmission rates.^5^ However, HF continues to have significant negative impacts on the quality of life of patients and their families or caregivers,^6^ remains a common cause of hospitalisation, and accounts for a substantial personal and economic burden.

Cardiac rehabilitation (CR) is a process by which patients with heart disease, in partnership with health professionals, are encouraged and supported to achieve and maintain optimal physical health.^7^ A recent Cochrane systematic review including 33 randomised trials in 4740 individuals with HF showed that participation in exercise-based CR was associated with a significant reduction in the risk of overall hospitalisation (relative risk: 0.75; 0.62 to 0.92, p=0.005) and HF-specific hospitalisation (relative risk: 0.61; 0.46 to 0.80, p=0.0004) and important improvements in patient health-related quality of life.^8^ Based on such accumulating evidence, in 2010 the UK National Institute of Health and Care Excellence (NICE) recommended offering CR based on supervised group exercise for patients with systolic and diastolic HF.^1^ Despite this recommendation, a survey in 2012 indicated that few UK centres (16% of those surveyed) had a specific rehabilitation programme for those with HF.^9^ The UK uptake of rehabilitation for people with HF therefore remains poor.^9^ A recent European survey on exercise training in HF concluded that ‘too many patients are still denied a highly recommended therapy’.^10^ We believe two key solutions to this poor provision and uptake are the development of a home-based self-help CR manual designed to meet the needs of those with HF and the close involvement of their caregivers.

The Rehabilitation EnAblement in CHronic Heart Failure (REACH-HF) research programme was designed to develop and evaluate a health professional facilitated home-based self-help manual rehabilitation intervention to improve self-care and health-related quality of life (HRQoL) in people with HF and their caregivers.

## Aims and hypothesis

This trial aims to assess the clinical effectiveness and cost-effectiveness of the addition of the REACH-HF intervention to usual care in patients with systolic HF and their caregivers. The primary hypothesis is that REACH-HF plus usual care (as received by participants in the ‘intervention group’) compared with usual care alone (as received by participants in the ‘control group’) can improve the disease specific HRQoL of patients at 12 months’ follow-up (primary outcome). Secondary objectives of the trial are:

- To compare secondary outcomes between patients in the intervention and control group (comprising the composite outcome of all-cause death or HF-related hospital admission, brain natriuretic peptide levels, exercise capacity, psychological well-being, level of physical activity, generic health-related quality of life and safety);
- To estimate the cost-effectiveness of the REACH-HF intervention plus usual care versus usual care alone, for patients with systolic HF;
- To explore the moderators and mediators of change in disease-specific HRQoL of patients in intervention and control groups;
- To assess the impact of, acceptability and satisfaction of the REACH-HF intervention to patients and caregivers;
- To compare psychological well-being, quality of life, self-care activities, and burden, between caregivers in the intervention and control groups;
- To check the fidelity of delivery of the REACH-HF intervention to patients and caregivers.

## Methods and analysis

This protocol is reported in accord with the Standard Protocol Items: Recommendations for Interventional Trials (SPIRIT) 2013 guidance for protocols of clinical trials.^11^

### Design

**Figure 1:**
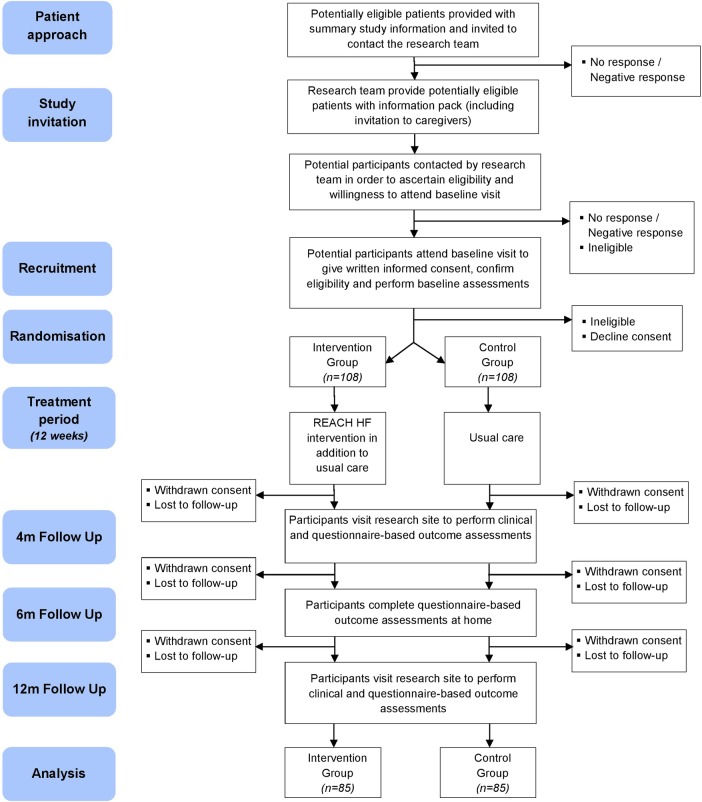
Illustration of study flow.

The study is a multicentre parallel two group randomised superiority trial with individual participant allocation to intervention group or control group with nested process and health economic evaluations. Given the complex nature of the intervention, it is not possible to blind participants or those involved in the provision of care. Researchers undertaking collection of outcome data and the statistician undertaking the data analysis will be blinded to treatment allocation in order to minimise potential bias. An illustration of the study flow is given in figure 1.

### Setting

The study will be conducted in four investigator centres in the UK: Birmingham (Sandwell and West Birmingham Hospitals NHS Trust), Cornwall (Royal Cornwall Hospitals NHS Trust), Gwent (NHS Wales) and York (York Teaching Hospital NHS Foundation Trust). Participants will be recruited at each of the four sites. To achieve adequate participant enrolment to sample size, each site can recruit through either primary or secondary care pathways, with each site having the opportunity to implement secondary strategies depending on recruitment performance which will be formally reviewed periodically by the central trial management team. Follow-up procedures will be conducted on NHS and non-NHS premises. Conduct of the study at each centre will be led by a local Principal Investigator supported by a research nurse(s) who has received training in Good Clinical Practice and in the requirements of the study protocol. Each participating site is responsible for the recruitment and scheduled follow-up visits of participants.

### Study population

The study population includes patients and caregivers. Participating patients will be aged 18 years or older and have a confirmed diagnosis of systolic HF on echocardiography or angiography (ie, left ventricular ejection fraction <45% within the past 5 years). Patients who have undertaken CR within 12 months prior to enrolment will be excluded, as will patients contraindicated to exercise testing or exercise training (adjudged according to adapted European Society of Cardiology guidelines for HF).^12^ The complete list of patient inclusion and exclusion criteria is provided in box 1.

Box 1 **Trial entry criteria**

Inclusion criteria

- Provision of informed consent to participate.
- Adults (aged ≥18 years).
- Patients who have a confirmed diagnosis of systolic heart failure (HF) on echocardiography (ie, left ventricular ejection fraction <45% within the past 5 years).
- Patients who have experienced no deterioration of HF symptoms in the past 2 weeks resulting in hospitalisation or alteration of HF medication

*Exclusion criteria*

- Patients who have undertaken cardiac rehabilitation (CR) within the past 12 months
- Patients who have received an intracardiac defibrillator (ICD), Cardiac Resynchronisation therapy (CRT) or combined CRT/ICD device implanted in the last 6 months.
- Patients who have any of the following contraindications to exercise testing or exercise training documented in their medical notes:
  Early phase after acute coronary syndrome (up to 2 days)
  Untreated life-threatening cardiac arrhythmias
  Acute HF (during the initial period of haemodynamic instability)
  Uncontrolled hypertension (systolic blood pressure >200 and/or diastolic blood pressure >100)
  Advanced atrioventricular block
  Acute myocarditis and pericarditis
  Symptomatic aortic stenosis
  Severe hypertrophic obstructive cardiomyopathy
  Acute systemic illness
  Intracardiac thrombus
  Progressive worsening of exercise tolerance or dyspnoea at rest over previous 3–5 days
  Significant ischaemia during low-intensity exercise (<2 Metabolic equivalents, <50 Watts)
  Uncontrolled diabetes (blood glucose >16 mmol/L or glycated hemoglobin >9% or equivalent unit)
  Recent embolism
  Thrombophlebitis
  New-onset atrial fibrillation/atrial flutter
- Patients who are in a long term care establishment or who are unwilling or unable to travel to research assessments or accommodate home visits.
- Patients who are unable to understand the study information or unable to complete the outcome questionnaires.
- Patients judged to be unable to participate in the study for any other reason (eg, psychiatric disorder, diagnosis of dementia, life threatening co-morbidity)
- Patients participating in concurrent interventional research which may over-burden the patient or confound data collection.

Participating caregivers will be aged 18 years or older and provide unpaid support to patients who could otherwise not manage without such support. Unpaid support includes emotional support, prompting with taking medications, observing for signs and symptoms of HF, getting prescriptions, encouraging participation in social events and physical activity, helping with household tasks or providing physical care.

A patient may still participate if s/he does not have an identified caregiver, or if the patient's caregiver is not willing to participate. Similarly patients who are unable or not willing to undertake the exercise capacity assessment will not be excluded.

Participants are free to withdraw from the study at any time, and this will be emphasised during the consent process. If a participant chooses to withdraw they will be asked to provide a reason and the reason for withdrawal will be noted. Participants do not have to provide a reason and this will be reiterated by the PI (or authorised delegate) in the event of a withdrawal request. Data collected on participants prior to withdrawal will be retained for analysis.

### Randomisation

Participants will be randomly allocated in a 1:1 ratio to either intervention or control group arms. Randomisation will be stratified by investigator site and baseline pro-brain natriuretic peptide (NT pro-BNP) levels (≤2000, >2000 pg/mL) using minimisation to facilitate balance between the two treatment arms. Randomisation numbers will be computer generated and assigned in strict sequence. At the point of randomisation, participants will be assigned the next randomisation number in the sequence. To maintain concealment and minimise selection bias, randomisation will be performed after the baseline visit by a member of Peninsula Clinical Trials Unit (CTU), independent from investigator teams, using a secure, web-based randomisation system.

### Intervention

The REACH-HF intervention is grounded in the support needs and priorities of people living with HF and the services that provide care for them. A systematic, six-step Intervention Mapping framework^13^ guided intervention development, drawing on research evidence, national and international guidelines and stakeholder consultations with patients, caregivers and health professionals to identify ‘targets for change’. In line with Intervention Mapping regulatory processes, underpinning target behaviour patterns and evidence-based change techniques were matched to each behaviour-change target.^14^ A key element of the intervention development process was an active Patient and Public Involvement group consisting of six people with a range of experiences with HF and three caregivers of people with HF. The intervention development process is described in detail elsewhere (CJ Greaves, C Deighan, P Doherty, *et al*. Development of a facilitated self-care and rehabilitation intervention for people with HF and their care givers: Rehabilitation Enablement in Chronic Heart Failure (REACH-HF). Submitted for publication 2015).

The REACH-HF intervention is a comprehensive self-care support programme comprising the ‘Heart Failure Manual’ (HF Manual), with a choice of two exercise programmes for patients, a ‘Family and Friends Resource’ for caregivers, a ‘Progress Tracker’ tool and a training course for intervention facilitators.

Participating patients and caregivers will work through the self-help manual over a 12-week period with facilitation by a specially trained intervention facilitator (cardiac nurse or physiotherapist by background), who will help to build the patient's and caregiver's understanding of how to manage HF. The manual includes information and interactive elements covering a wide range of topics relating to living with/adapting to living with HF, and includes four core elements:

1. An exercise training programme, tailored according to initial fitness assessments, delivered as a walking programme or a chair-based exercise DVD, or a combination of the two (the patient's choice);
2. Managing stress /breathlessness /anxiety;
3. HF symptom monitoring (and associated help-seeking);
4. Understanding and taking medications.

Patients will be encouraged to use the progress tracker booklet, which is designed to collect the following information over the period of the intervention: changes in physical and mental state, intensity of exercise and self-reported walking speed, and degree of completion of self-monitoring sections for physical activity, enjoyable activities, frequency of self-weighing (to monitor fluid build-up), and frequency of self-reported use of stress-management techniques. The Family and Friends resource, a manual for use by caregivers, includes advice on providing support, becoming a caregiver, managing caregiver's own health and well-being and getting help.

As a pragmatic trial of a self-help intervention which is reliant on the willing engagement of recipients, there are no specific strategies to improve participants’ adherence to intervention protocol. Intervention delivery may be discontinued at any time at the request of a participant or by the intervention facilitator if they determine that the intervention may be the cause of undue harm.

Adherence to intervention protocols from the perspective of the intervention facilitators will be ascertained through fidelity assessment described herein.

### Usual care

In accord with findings of our national survey,^9^ patients with HF typically do not receive CR, despite NICE recommendations.^1^ The choice of a usual care (no rehabilitation) comparator in the REACH-HF trial is therefore reflective of the situation for the vast majority of patients with HF. In this trial, intervention and control group patients will receive usual medical management for HF according to national and local guidelines, including specialist HF nurse care. The use of care services, including those provided by specialist HF nurses in the community and in secondary care, will be documented at each follow-up through participants’ completion of healthcare resource use questionnaires and by collection of concomitant medication usage as reported by participants.

### Outcome measures

Outcome data will be collected at 4, 6 and 12 months following the baseline visit (table 1—Tabulated summary of study schedule). The 4-month time point coincides with the end of the 3-month intervention delivery period for participants in the intervention arm. This allows a 1 month period after the baseline visit for completion of randomisation and referral processes.

**Table 1:** Tabulated summary of study schedule

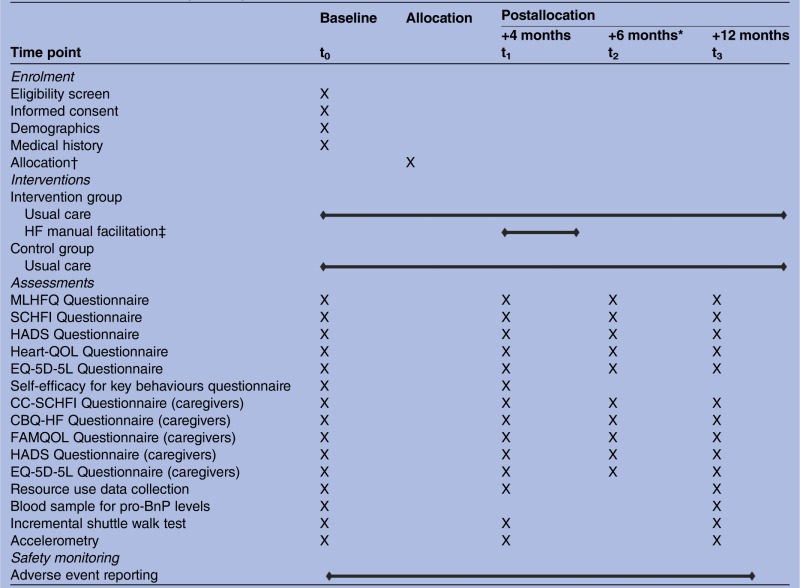

*Six month time point is conducted by post. Participants are not required to visit the research centre at this time point.

†Allocation will be performed by Peninsula Clinical Trials Unit (CTU), typically within 10 days of the baseline clinic, following receipt of baseline data and blood sample result.

‡HF Manual f*a*cilitation will commence approximately 1 month post randomisation.

HADS, Hospital Anxiety and Depression Scale questionnaire; HF, heart failure; MLHFQ, Minnesota Living with HF questionnaire; pro-BNP, pro-brain natriuretic peptide; QoL, quality of life; SCHFI, Self-care of HF Index questionnaire; CC-SCHFI, Caregiver Contribution to Self-care of HF Index questionnaire; CBQ-HF, The Caregiver Burden Questionnaire for HF; FAMQOL, Family Caregiver QoL questionnaire.

#### Primary outcome

Patient disease-specific HRQoL measured using the Minnesota Living with HF questionnaire (MLHFQ) at 12 months. The questionnaire consists of 21 items and is designed to represent the ways HF and treatments can affect the key physical, emotional, social and mental dimensions of an individual's quality of life.^15^

#### Secondary outcomes

##### Patients

- Composite outcome of death or hospital admission related to HF or not related to HF. All instances of hospitalisation and death will be recorded and made accessible to an independent adjudication panel of three experienced cardiologists who will ascertain whether or not reported events are HF-related.
- NT pro-BNP levels. Natriuretic peptide levels are elevated in patients with HF.^16^
- Exercise capacity (incremental shuttle walk test (ISWT)).^17^
- Physical activity level (accelerometry over a 7-day period, measured using the GENEActiv Original accelerometer).^18^
- Psychological well-being using Hospital Anxiety and Depression Scale questionnaire (HADS).^19^
- Generic HRQoL using the EQ-5D-5L questionnaire.^20^
- Disease-specific quality of life using the Health-related Quality of Life (HeartQoL) questionnaire.^21^
- Self-care of HF Index questionnaire (SCHFI).^22^
- Healthcare utilisation (ie, primary and secondary care contacts, social care contacts and relevant medication usage).
- Self-efficacy for key behaviours questionnaires (developed by the research team).
- Safety; recording and reporting of serious adverse events. Any adverse event or adverse reaction will be regarded as serious if it: results in death, is life-threatening, requires hospitalisation or prolongation of existing hospitalisation, results in persistent or significant disability or incapacity. All serious adverse events that occur during the trial will be recorded and reported to the Ethics Committee, the Data Monitoring Committee and the Trial Steering Committee.

##### Caregivers

- Psychological well-being using the HADS.^19^
- Generic HRQoL using the EQ-5D-5L questionnaire.^20^
- Caregiver Contribution to Self-care of HF Index questionnaire (CC-SCHFI).^22^
- Caregiver Burden Questionnaire—HF.^23^
- Family Caregiver Quality of Life Scale questionnaire (FAMQOL).^24^

### Sample size

The sample size is based on an effect size that represents a clinically important difference and is plausible. The developers of the MLHFQ have determined that five points is the minimal clinically important difference in score.^15^ With a type I error of 0.05 and power of 90%, 85 patients per group are required to detect a five-point difference in the MLHFQ score, assuming a SD of 10.^25^ With an attrition rate of 20% (in accordance with the level of attrition seen in previous trials),^26^ ^27^ 108 patients are required per group. The plausibility of this between group difference is supported by the Cochrane review of CR in HF, which reported a mean pooled between group difference of 10.3 (95% CI 4.8 to 15.9) points in MLHFQ score.^28^ The proposed sample size is likely to be conservative given the analysis of covariance approach to primary outcome analysis and adequate to detect an important difference in a number of secondary outcomes, including the ISWT (50 m) and Hospital Anxiety and Depression Scale (1.5 points) at a power of 80% or higher.

### Trial data collection

Trial data are collected from participants during three clinic visits (at baseline, 4 and 12 months) and by postal questionnaire at 6 months. To encourage participant retention and completeness of data, participants may claim travel expenses associated with clinic visits and are provided with postage-paid envelopes to return questionnaires by post. Furthermore, visits may be partially conducted at participants’ homes if mutually convenient for the research nurse and participant. Participants who are unwilling or unable to travel to research assessments or accommodate home visits are excluded at the point of consent.

At the baseline clinic visit, after written informed consent has been obtained by the research nurse, the following information will be collected:

- Medical history (including comorbidities (number and severity scored with Charlson Co-morbidity Index), New York Heart Association class, HF aetiology, concomitant HF medication and presence of implantable HF devices);
- Healthcare resource utilisation over the prior 6 months;
- Sociodemographic information (ie, date of birth, ethnicity, height, weight, employment status, education level, smoking status).

Participating patients will be asked to:

- Complete a booklet comprising the primary and secondary outcome questionnaires;
- Perform an the incremental shuttle walk test;
- Provide a (∼4 mL) blood sample for measurement of NT pro-BNP levels;
- Wear a wrist-worn accelerometer for 7 days.

Participating caregivers will also be asked to provide sociodemographic information (ie, date of birth, ethnicity, weight, employment status, education level and smoking status) and to complete a booklet comprising their outcome questionnaires.

Patient and caregiver follow-up outcome assessments will be performed at clinic visits held at 4 and 12 months after the baseline visit, with a postal follow-up (questionnaire-based outcomes only) performed at the 6-month time point. At the 4 and 12-month clinic visits investigators will record details of any changes to participants’ HF medication or implantable cardiac devices, details of any hospitalisations and healthcare resource utilisation since the previous visit. Investigators will also check that participating patients have not become contraindicated to exercise testing before conducting the incremental shuttle walk test. Blood samples (collected at baseline and 12 months only) will be dispatched to a central laboratory (Royal Cornwall Hospital NHS Trust) for determination of NT pro-BNP levels. Accelerometer devices will be returned by participants using postage-paid envelopes after 7 days of wearing. The devices will be returned to the CTU for data extraction. Participant safety will be monitored through recording, reporting and review of all serious adverse events collected from baseline until final follow-up visit.

Data collected at clinic visits will be recorded on study specific case report forms (CRFs) by the research team at each site. Completed CRFs will be checked and signed at the research sites by a member of the research team before being sent to the CTU. Original CRF pages and completed questionnaire booklets will be posted to the CTU at agreed time points for double-data entry in to the study database. Accelerometer data will be imported directly into the study database. All forms and data will be tracked using a web-based trial management system. Double-entered data will be compared for discrepancies according to a data management plan held in CTU. Discrepant data will be verified using the original paper data sheets.

Participant names and addresses will be collected for the purpose of managing questionnaire dispatch, intervention delivery and participant interviews. Investigators will ensure that the participants’ anonymity is maintained on all other documents. Within the CTU, anonymised and identifiable study data will be stored separately, to prevent the identification of participants from research records, in locked filing cabinets within a locked office. Electronic records will be stored by the CTU in a web-based, SQL server database, housed on a restricted access, secure server maintained by the University of Plymouth. Data in the database will be backed up daily. The website will be encrypted using SSL. Data will be collected and stored in accordance with the Data Protection Act 1998. Direct access to the trial data will be restricted to members of the research team and the CTU, with access granted to the Sponsor on request. Access to the database will be overseen by the CTU data manager and trial manager.

### Process evaluation

The process evaluation seeks to assess intervention fidelity, patients' and caregivers' experiences of trial participation, and to explore processes that may be responsible for change in the primary outcome of HRQoL (including intermediate changes in secondary outcomes and changes in self-care behaviour patterns of patients receiving the REACH-HF intervention).^29^

Patient and caregivers’ views on the intervention will also be explored as part of the process evaluation. Five distinct studies comprise the process evaluation as follows:

#### Process evaluation study 1: Intervention fidelity

A fidelity checklist developed and piloted as part of the REACH-HF programme (CJ Greaves, *et al* Submitted for publication 2015) will be used to assess fidelity of delivery of the intended intervention processes. This will be achieved by analysing recordings of all contacts (telephone and face to face) between intervention facilitators and 20 purposively sampled patient participants. Contacts will be audio recorded by intervention facilitators and the files made accessible to two researchers who were part of the REACH-HF intervention development team (a chartered health psychologist and a registered nurse (by background)) who will complete the checklist while listening to the recordings. The check list is based on the Dreyfus scale.^30^ This will clarify how well (or otherwise) intervention components are delivered and received and will also allow researchers to describe variability in fidelity of delivery across patients and facilitators.

#### Process evaluation study 2: Experiences of patients

Interviews with each of 20 patients selected above will be conducted immediately after completion of intervention delivery and again at 12 months after the baseline visit. The interviews will be audio recorded and transcribed verbatim. Interviews will be conducted according to topic guides covering patients’ engagement with the intervention, their relationship with their facilitator, involvement of family and friends, use of the manual, behavioural change and psychological adjustments to living with HF.

#### Process evaluation study 3: Experiences of caregivers

Interviews with up to 20 purposively sampled caregivers (including caregivers of patients participating in Study 1) will be conducted at 4 and 12 months. Where possible, the patient and the caregiver will be interviewed separately. Topics covered in the interviews will include the caregiver's role before participating in the REACH-HF trial, their engagement with the intervention, the impact of the intervention, the caregiver's relationships with the patient and the facilitator and the ways in which the caregiver has adjusted his/her behaviour as a result of the intervention. The researcher leading the caregiver interviews will work closely with the researcher conducting the patient interviews and will review the topic guide throughout the study so that questions are informed by relevant emerging topics. Interviews will be audio recorded and transcribed verbatim.

#### Process evaluation study 4: Identification of potential outcomes as mediators of effectiveness

Observed differences in secondary outcomes at intermediate follow-up points (4 and 6 months) provide an indicator of change for participants in the intervention and control groups. Such changes may be predictive of the primary outcome of MLHFQ at 12 months. Potential intermediate outcomes that may be considered include exercise capacity, psychological well-being, physical activity and self-efficacy for key behaviours including physical activity.

#### Process evaluation study 5: Use of progress trackers to identify patient changes associated with effectiveness

As described earlier, the REACH-HF Manual includes a progress tracker which intervention patients will be encouraged to use to track their progress including physical activity, mood, symptoms and self-care actions. At the end of the intervention delivery period, copies of the participants’ progress trackers will be provided to the research team and digitally scanned. This information together with the number of facilitator contacts and total contact time received, will allow characterisation of individual patient engagement in the intervention. To the extent that this is the case, tracker scores can be used to explore potential changes which indicate likelihood of intervention success.

### Economic evaluation

An economic evaluation will be undertaken to estimate the cost-effectiveness of the REACH-HF intervention plus usual care versus usual care alone in patients with systolic HF. Cost-effectiveness analyses will be undertaken using clinical and resource-use data collected within the trial over a 12-month time horizon. The primary perspective will be that of the UK NHS and Personal Social Services, with a broader perspective, addressing partial patient and societal perspective, considered in sensitivity analyses. The primary economic end point will be the quality-adjusted life-year (QALY), using the EQ-5D-5L, over the 12-month follow-up. The economic evaluation will estimate the incremental cost per QALY associated with the REACH-HF intervention.

The additional (incremental) costs associated with delivery of the HF Manual, when added to usual care, will be estimated using resource use data collected within-trial, and unit costs for resource use from national published or NHS sources. Resource use is expected to consist of time input from REACH-HF facilitators, supervision for facilitators, training costs for facilitators and consumables (eg, booklets). Data on facilitator time input will be captured via facilitator self-report within trial at participant level, using purpose-designed forms.

Health, social care and other resource use data will be collected within trial at participant level and are collectively regarded as a secondary outcome measure. Resource use data will be used in combination with unit costs to compare health, social care and other resource use between groups, as perspective employed. Data will be collected from participants by self-reported (interviewer administered) participant questionnaire at baseline, 4-month and 12-month time points. Hospitalisation data (events) will be collected as part of ‘adverse event’ reporting and HF related medication data as reported by patients will be captured by the research nurse at the research clinics.

### Data analysis

#### Primary and secondary outcomes

All analyses, quantitative and qualitative, will be conducted according to best practise and reported in accordance with Consolidated Standards of Reporting Trials (CONSORT) guidelines for reporting of clinical trials^31^ and appropriate guidelines for reporting process evaluations^32^ and qualitative research.^33^ Baseline sociodemographic and health-related variables will be reported descriptively by treatment arm, in order to assess whether the inferential analyses require adjustment for any unbalanced variables.

The primary analyses for all patient and caregiver outcomes will be based on a between-group, intention-to-treat, complete case approach, using data collected at 12 months’ follow-up. The outcomes will be analysed using the regression method appropriate to the data, that is, linear regression modelling for continuous outcomes, survival analysis based on the Cox proportional hazards regression model for time-to-event data, and Tobit regression analysis for EQ-5D-5L. All analyses will adjust for baseline score of the outcome variable (where applicable), as well as minimisation variables previously described, and sociodemographic and health-related variables that are found to be unbalanced at baseline.

Secondary analyses will be undertaken on patient and caregiver outcomes as repeated measures analysis using all follow-up assessment points (4, 6 and 12 months). In addition, a per protocol analysis of the primary outcome will be performed using 12-month follow-up data. A per protocol definition (based on a minimum level of intervention uptake and adherence deemed necessary to achieve improvement in outcomes) will be agreed prior to the start of data analysis. If there is more than 5% loss to follow-up for the primary outcome at 12 months, multiple imputation methods will be used as a sensitivity analysis to address the issue of missing data. The following subgroups will be assessed: the stratification variables of trial centre and severity of HF (NT pro-BNP levels), plus time since HF diagnosis and the inclusion (or not) of a caregiver.

The potential for differential intervention effects within patient subgroups (ie, moderation by patient characteristics) will be explored using interactions within linear regression modelling for the primary outcome only. Mediation analyses will be used to assess the extent to which secondary outcomes at intermediate follow-up (eg, self-efficacy or physical activity levels at 4 months) or progress tracker self-care behaviours (eg, self-reported exercise, stress or anxiety management activities) can explain between-group differences in the primary outcome at 12 months. Moderation and mediation analyses will be exploratory in nature as no formal power calculation for interaction effects has been performed.

Serious adverse events will be presented descriptively by treatment arm.

All between group outcome results will be presented as means and 95% CIs. No correction of p values for multiplicity of testing will be undertaken. However, the primary outcome (MLHFQ at 12 months) analysis will be performed before all other analyses and the p values of all subsequent analyses interpreted in the context of multiple testing. No interim analyses will be performed. All analyses will be conducted by a statistician who is blinded to treatment arm, using Stata V.12.

#### Economic outcomes

Means (and SDs) for resource use and costs will be presented for baseline assessment, and for resource use over the 12-month follow-up period. Regression methods will be used to estimate mean costs per group and to compare mean costs between treatment and control groups. MLHFQ data will be drawn from the main statistical analyses. QALY data will be derived from trial data on EQ-5D-5L, using a UK algorithm/tariff, in the first instance those derived from Dolan^34^ (via van-Hout *et al*^35^; although it is expected that a UK tariff will be published at the time of analysis), for the 5-level EQ-5D). Derived health state values will be used to estimate QALYs through application of standard area-under-the-curve methods^36^ using all data from baseline to 12-month. Analysis of mean QALY per group, and differences between groups will be undertaken using regression based methods, adjusting for baseline EQ-5D-5L, and using covariates as the main statistical analyses on effectiveness. As analyses are over a 12-month period no discounting of (future) costs or outcomes is required.

#### Qualitative outcomes

A thematic analysis of interviews will be conducted^37^ ^38^ to generate emerging themes and overarching themes.^37^ Other members of the team will conduct independent analyses of subsets of the data, and the qualitative team will meet regularly to discuss coding and analysis. Reflexive notes will also be used to help assure transparency and trustworthiness of the analysis.^39^ The analysis will characterise patients’ observed and self-reported responses to the intervention and link these responses to overall use and perceived benefit, identifying interpersonal and intrapersonal processes that shape effectiveness or ineffectiveness of the intervention. At 4 months, patients’ engagement with, response to and use of the REACH-HF Manual will be characterised and differences between patients noted. At 12 months overall use of and benefit derived from the REACH-HF manual, and maintenance of self-care behaviours and coping skills will be characterised and linked to individual differences in 4-month responses. This will allow a qualitative description of potential pathways and barriers to improvement. Data from the caregiver interviews will be analysed using similar methods.

### Data monitoring and quality assurance

The Site Principal Investigators (JA, HD, PD, RD, KJ, RVL) or authorised delegate will check completed CRFs for missing data or obvious errors before the forms are sent to the CTU. Data will be monitored centrally for quality and completeness by the CTU and every effort will be made to recover data from incomplete forms where possible. The CTU data manager will oversee data tracking and data entry and initiate processes to resolve data queries where necessary. The CTU trial managers (CH and VE) will devise a monitoring plan specific to the study which will include central monitoring strategies and study site visits as appropriate. Participating sites will be required to permit the CTU trial manager or deputy, or representative of the sponsor, to undertake study-related monitoring to ensure compliance with the approved study protocol and applicable standard operating procedures (SOPs), providing direct access to source data and documents as requested. All study procedures will be conducted in compliance with the protocol and according to the principles of the International Conference on Harmonisation Good Clinical Practice (ICH GCP). Procedures specifically conducted by the CTU team (eg, data management, study management and study monitoring) will be conducted in compliance with CTU SOPs.

### Trial management and independent committees

Team members directly involved with the day-to-day running of the trial will meet weekly to discuss trial progress, teleconferencing with site PIs on a monthly basis with email and telephone exchange as necessary between. The Programme Management Group including health economics, statistics, process evaluation and patient and public representation will meet on a termly basis to review status of the overall programme, including trial progress.

The REACH-HF Programme Steering Committee (Chair: Professor Martin Cowie and four other independent members including a patient and public involvement representative) have formally agreed to adopt the role of Trial Steering Committee and will oversee the conduct of the trial with safety and ethics review by a fully independent Data Monitoring Committee (Chair: Dr Ann-Dorthe Zwisler and two other independent members). Evidence for treatment differences in the main efficacy outcome measures will not be monitored through review of accumulating outcome data and no interim data analyses will be conducted.

The Trial Steering Committee and Data Monitoring Committee meet one to two times per year. Detailed descriptions of the remit and function of the oversight committees are documented in specific charters held in the Trial Master File by CTU.

## Ethics and dissemination

The study will be conducted in accordance with the ethical principles that have their origin in the Declaration of Helsinki and that are consistent with ICH GCP, and in accordance with the Research Governance Framework for Health and Social Care, Second edition (2005). The study is sponsored by Royal Cornwall Hospitals NHS Trust (Research, Development & Innovation Department, Royal Cornwall Hospital, Treliske, Truro, Cornwall, TR1 3LJ). Written informed consent will be obtained from all participants prior to study enrolment. Participants enrolled into the study are covered by indemnity for negligent harm arising from the management, design and conduct of the research through standard NHS Indemnity arrangements. The study is approved by the National Research Ethics Service Committee North West—Lancaster Research Ethics Committee (reference 14/NW/1351). Any subsequent amendments will be made using the Integrated Research Applications System in order to maintain ethical approval and NHS permissions. Amended documents will be provided to investigator sites by CTU. In the event of changes to study design requiring significant amendment to the content of the participant information sheet, participants will be required to provide renewed informed consent.

Findings will be published in peer-reviewed journals and presented at local, national and international meetings and conferences to publicise and explain the research to clinicians, commissioners and service users. A final report will be submitted to the National Institute for Health Research and a summary report will be circulated to NHS commissioners and service providers, patient groups and trial participants. All investigators will have access to the final data set. Participant-level data sets will be made accessible on a controlled access basis.

## Conclusion

This randomised controlled trial aims to assess the clinical and cost-effectiveness of the Rehabilitation Enablement in Chronic Heart Failure (REACH-HF) intervention, a manualised home-based rehabilitation intervention designed to improve self-care and HRQoL in people with systolic HF. We will also assess the outcomes of caregivers. The study results will provide valuable information for clinicians, policymakers, patients and their caregivers about the role of self-directed rehabilitation interventions and has the potential to positively impact on the current dearth in the provision and uptake of rehabilitation services for people with HF and caregiver support.
